# Volume-dependent analgesia and dermatomal regression during continuous rhomboid intercostal plane block: lessons from a multiple rib fracture case in intensive care unit

**DOI:** 10.1186/s12871-025-03344-z

**Published:** 2025-10-14

**Authors:** Tolga Karaçay, Başak Altıparmak, Canan Gürsoy, Melike Korkmaz Toker

**Affiliations:** Department of Anesthesiology and Reanimation, Muğla Sıtkı Koçman University Faculty of Medicine, Muğla, Türkiye Turkey

**Keywords:** Acute pain, Interventional pain management, Pain outcome measurement, Postoperative pain, Ultrasound in pain medicine, Regional anesthesia

## Abstract

**Background:**

Multiple rib fractures can cause severe respiratory issues if not managed with early and adequate pain control. Without timely and appropriate analgesia, these fractures may lead to significant respiratory complications. Interfascial plane blocks, such as the Rhomboid Interfascial Plane block (RIB), serve as effective adjuncts to pharmacological agents like opioids. However, there is limited literature on the optimal anesthetic volumes and dermatomal spread associated with Rhomboid Interfascial Plane catheters.

**Case presentation:**

A 52-year-old male involved in a motor vehicle accident was diagnosed with non-displaced fractures of ribs 4–9. He was monitored in the intensive care unit, and a Rhomboid Intercostal Plane block was performed at the T6–T7 level using 30 mL of 0.25% bupivacaine. A catheter was then placed at the same level for continuous analgesia.

**Conclusions:**

The Rhomboid Interfascial Plane catheter provided effective pain relief. Over a 24-hour follow-up period, a reduction in dermatomal spread was noted, underscoring the need for further research into optimal dosing and catheter placement techniques for patients with multiple rib fractures.

## Background

Multiple rib fractures are a serious medical condition that can occur after severe blunt thoracic trauma. Poorly managed pain from rib fractures can lead to respiratory problems, increasing both morbidity and mortality. Insufficient ventilation, difficulty coughing, and inadequate clearance of secretions may cause complications such as atelectasis and pneumonia [1].

Several studies have shown that thrombocytopenia is common among critically ill patients, with prevalence rates ranging from 20 to 40% upon ICU admission and similar rates of incident thrombocytopenia during their stay. Thrombocytopenia in this group has been linked to higher morbidity and mortality, making it an important factor to consider when planning regional anesthesia. Although thrombocytopenia represents a critical concern in intensive care unit, interfascial plane blocks can be safely performed in these patients [2–4].

The Rhomboid Intercostal Block (RIB) has been employed as an effective method for pain control following breast surgery, thoracic surgery, and rib fractures [5–7]. When performed under ultrasound guidance at the T6–T7 vertebral level, local anesthetic spreads within the fascial plane between the rhomboid muscle and the ribs, providing analgesia over the T3–T8 dermatomes. Some clinicians have reported that the analgesic effect may extend to the T9 level. In certain cases, the Sub-serratus Plane Block (SSP) is added to the RIB to achieve analgesia at lower thoracic levels [8, 9]. However, each additional intervention introduces a new potential risk of complications such as hemotorax, pneumothorax, vascular injection local anesthetic systemic toxicity (LAST), etc.

In this report, we present a case of a patient with multiple rib fractures extending to the T9 level, in whom effective pain palliation and assessment of the volume–dermatome relationship were achieved using RIB block and catheter placement alone, without the addition of an SSP block.

## Case presentation

A 52-year-old male patient with a medical history of hypertension, type 2 diabetes mellitus, and heart failure, American Society of Anesthesiologists Physical Status 3, and a body mass index (BMI) of 29 kg/m², was admitted to the emergency department following a motor vehicle accident. The patient had no prior history of anticoagulant therapy and no evidence of coagulopathy. Imaging with Computer Tomography revealed a burst fracture at the T7 vertebral level accompanied by non-displaced rib fractures between the 4th and 9th ribs. Due to associated mesenteric injury, splenic laceration, and tachypnea, the patient was transferred to the intensive care unit (ICU) for further monitoring. In the ICU, pain was assessed using the Numeric Rating Scale (NRS). The patient reported a score of 10/10 in the left posterior hemithorax between the T4–T9 dermatomes, both at rest and during coughing. To achieve effective analgesia, a RIB was planned, along with the placement of a catheter for continuous local anesthetic infusion.

**Fig. 1:**
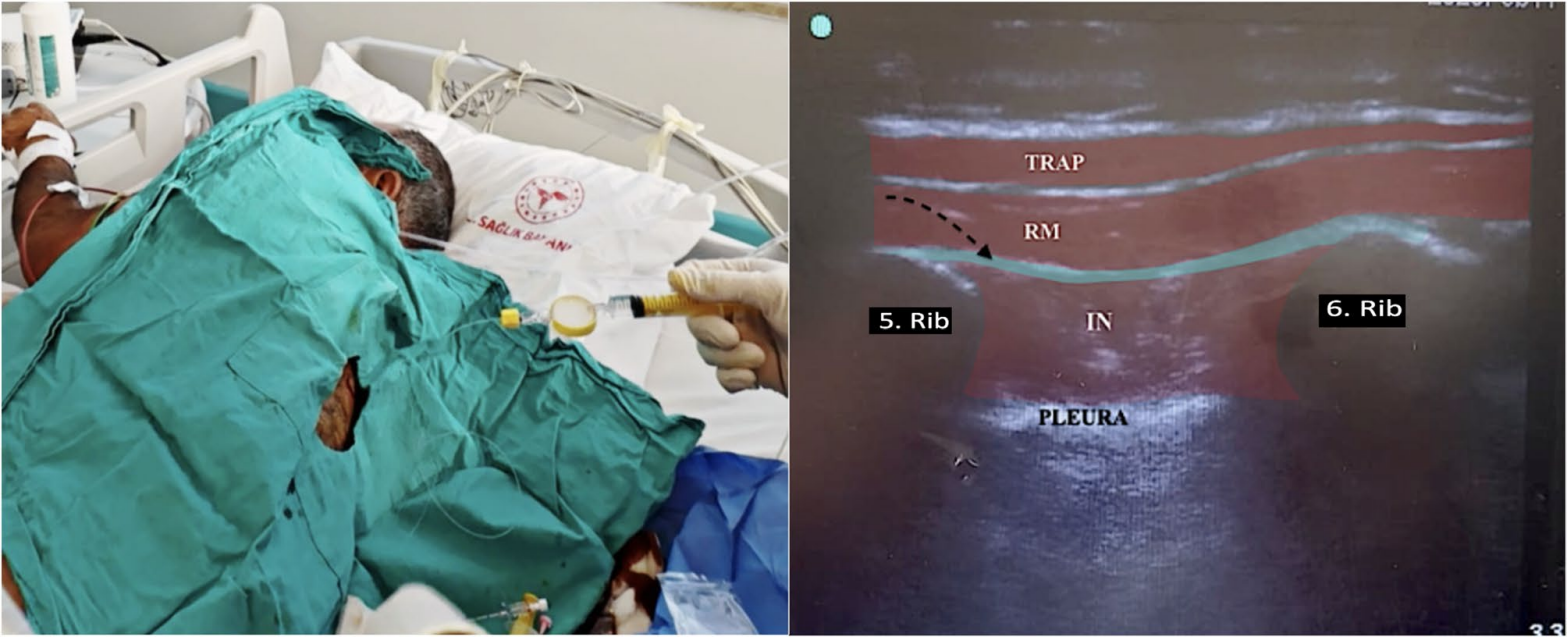
Ultrasound guided rhomboid intercostal plane block and placement catheterization. TRAP: Trapezius muscle RM: Rhomboid muscle IN: Intercostal muscle

The patient was hemodynamically stable, was positioned in the lateral decubitus position with the left arm crossed over the chest. Due to rib fractures and associated tachypnea (peripheral oxygen saturation: 90%), no sedation was administered. Intravenous paracetamol (1000 mg) was initiated as an infusion. After skin sterilization with povidone iodine and lidocaine infiltration at the injection site, a linear ultrasound probe was placed at the T6–T7 level. A block needle was directed into the interfascial plane between the rhomboid major and intercostal muscles. A total of 30 mL of 0.25% bupivacaine was injected, followed by placement of a catheter at the same level (Fig. 1). Twenty minutes after the procedure, the NRS score during coughing decreased to 3/10. A dermatome analysis using the pinprick test performed at the second hour revealed sensory blockade from T3 to T9. At this point, the NRS score was 0 at rest and 3 during coughing. Continuous infusion of 200 mg bupivacaine diluted in 500 mL isotonic solution was initiated via a patient-controlled analgesia (PCA) device at a basal rate of 20 mL/hour. PCA settings included a lock-out interval of 30 min and a 10 mL bolus dose. As part of multimodal analgesia, paracetamol was administered at a dose of 1 g three times daily.

Pain assessments using NRS were conducted at 20 min, and at 2, 4, 6, 8, 16, and 24 h post-procedure. NRS scores and dermatomal levels within the first 24 h are summarized in (Table 1). Although pain scores remained below 3/10 during both rest and coughing in the first 4 h, a dermatomal assessment at the 6th hour showed regression of the lower sensory level to T7, and pain scores between T7 and T9 increased to 3–4/10. A bolus of 10 mL 0.25% bupivacaine was administered via the PCA, and the settings were adjusted. To remain within the maximum safe dose of bupivacaine (3 mg/kg/day), the infusion solution was reformulated as 100 mg of bupivacaine in 500 mL isotonic solution (i.e., 0.0625% concentration). The basal infusion rate was increased to 30 mL/hour, and the bolus option was disabled. Approximately 30 min later, the patient reported marked pain relief. At subsequent time points, the NRS scores were recorded as 0 at rest and 2 during coughing, with dermatomal spread extending from T3 to T9. No additional rescue analgesic was required during the first 24 h following the procedure.

**Table 1:** The patient’s NRS scores and dermatomal effects at rest and during coughing in the first 24 hours

NRS	20. minute	2. hour	4. hour	6. hour	8. hour	16. hour	24. hour
Resting	0	0	0	0	0	0	0
During Coughing	3	3	3	4	2	2	2
Dermatomal Effect	T3-T9	T3-T9	T3-T9	T3-T7	T3-T9	T3-T9	T3-T9

*NRS* Numeric Rating Scale

## Discussion & Conclusion

In the palliative management of pain associated with multiple rib fractures, pharmacologic agents such as opioids, nonsteroidal anti-inflammatory drugs, ketamine, and gabapentinoids can be employed. In addition, regional anesthesia techniques including thoracic epidural analgesia, paravertebral block, serratus plane block and erector spinae plane block have gained increasing popularity [3]. The widespread use of regional techniques is partly due to the complications associated with systemic opioid use, such as sedation, suppression of the cough reflex, respiratory depression, and hypoxia [10].

Interfascial plane blocks and paravertebral block are commonly used for rib fracture pain relief. However, in critically ill patients with coagulopathy or who are on anticoagulants, invasive techniques like paravertebral blocks carry higher risks. Interfascial plane blocks are increasingly used as alternatives to paravertebral block, such as serratus anterior plane block [11], erector spinae plane block [12], and RIB [7]. In our clinical practice, we prefer the RIB block due to our greater experience with this technique.

Despite continuous infusion of local anesthetic via PCA, a regression of the block to more cranial dermatomes was observed in the later hours, resulting in pain in the lower thoracic levels. This finding suggested that, to maintain effective analgesia over time, an increase in the volume of local anesthetic may be required. There are limited studies in the literature analyzing the volume–dermatome relationship in RIB. One study using 25 mL of local anesthetic solution reported analgesia between the T2 and T9 dermatomes; however, follow-up dermatome analysis over time was not conducted [8]. Similarly, a cadaveric study of the Rhomboid Intercostal and Sub-serratus Plane (RISS) block performed with 30 mL of injectate demonstrated dye spread between T3 and T9 [9]. It is well established that increasing the injectate volume in interfascial plane blocks results in a broader spread. In the cadaveric study conducted by Çiftçi et al. [11] evaluating the Erector Spinae Plane Block, increasing the injectate volume from 20 mL to 30 mL resulted in a wider cranio-caudal spread. In our patient, to avoid LAST and complications associated with repeated injections, we chose to perform RIB with catheter placement rather than adding RISS block. Although the initial 30 mL dose provided analgesia from T3 to T9, a continuous infusion of 20 mL/hour became insufficient over time. To address this, we increased the infusion rate to 30 mL/hour while reducing the concentration of the local anesthetic to remain within the safe dosing limits. In a study by Wang et al. [12], hydrodissection of the myofascial plane alone resulted in meaningful pain relief, suggesting that the analgesic effect may not rely solely on local anesthetics. Based on this rationale, we diluted our injectate from 0.0125 to 0.00625% bupivacaine, aiming to maximize volume and tissue spread while minimizing the risk of toxicity. This approach is not expected to reduce efficacy, but rather represents a practical means of leveraging both the mechanical effect of hydrodissection and the pharmacologic contribution of local anesthetics. This approach resulted in improved analgesia in the later hours, with pain scores decreasing, dermatomal coverage returning to the original levels, and no need for rescue analgesics.

Although no complications related to RIB have been reported in the current literature, the potential risks of LAST, hematoma formation at the needle site, pleural puncture, and inadequate analgesia should always be considered, and unnecessary injections should be avoided.

In conclusion, although there have been previous case reports of rib fractures associated with the RIB, our observation regarding the infusion spreading below the intended dermatome during PCA administration is novel. Single-shot RIB followed by catheter placement and continuous infusion using a low-concentration, high-volume local anesthetic solution guided by close dermatome monitoring may represent an effective approach for managing acute pain due to multiple rib fractures. This technique may help optimize analgesia while minimizing systemic side effects and complications.

## Data Availability

The datasets used and/or analysed during the current study are available from the corresponding author on reasonable request.

## Abbreviations

BMI: Body mass index
ICU: Intensive care unit
LAST: Local anesthetic systemic toxicity
NRS: Numeric rating scale
PCA: Patient-controlled analgesia
RIB: Rhomboid interfascial plane block
RISS: Rhomboid intercostal and subserratus plane block
SSP: Subserratus plane block
